# A Design of Experiment (DoE) Approach to Model the Yield and Chemical Composition of Ajowan (*Trachyspermum ammi* L.) Essential Oil Obtained by Microwave-Assisted Extraction

**DOI:** 10.3390/ph14080816

**Published:** 2021-08-19

**Authors:** Eugenia Mazzara, Serena Scortichini, Dennis Fiorini, Filippo Maggi, Riccardo Petrelli, Loredana Cappellacci, Giuseppe Morgese, Mohammad Reza Morshedloo, Giovanni Filippo Palmieri, Marco Cespi

**Affiliations:** 1 School of Pharmacy, University of Camerino, 62032 Camerino, Italy; eugenia.mazzara@unicam.it (E.M.); filippo.maggi@unicam.it (F.M.); riccardo.petrelli@unicam.it (R.P.); loredana.cappellacci@unicam.it (L.C.); giuseppe01.morgese@studenti.unicam.it (G.M.); gianfilippo.palmieri@unicam.it (G.F.P.); 2 School of Science and Technology, University of Camerino, 62032 Camerino, Italy; serena.scortichini@unicam.it (S.S.); dennis.fiorini@unicam.it (D.F.); 3 Department of Horticultural Science, Faculty of Agriculture, University of Maragheh, Maragheh 83111-55181, Iran; morshedlooreza@gmail.com

**Keywords:** *Trachyspermum ammi*, essential oil, thymol, microwave-assisted extraction, fractional factorial design, central composite design

## Abstract

Ajowan (*Trachyspermum ammi* L.) is a spice traditionally used in Middle Eastern medicine and contains a valuable essential oil (EO) exploited in different fields, such as pharmaceutics, agrochemicals and food additives. This EO is mostly characterized by the thymol to which most of its biological properties are related. Given the economic value of ajowan and its increasing demand across the globe, the extraction method used for its EO is of paramount importance in terms of quality and quantity of the final product. In the present study, we used the design of experiment (DoE) approach to study and optimize the extraction of the ajowan EO using the microwave-assisted extraction (MAE), a novel extraction technique with high efficiency, low energy consumption, short process length and low environmental impact. A two-step DoE (screening followed by surface response methodology) was used to reduce the number of experiments and to improve the cost/benefit ratio. Reliable mathematical models, relating the more relevant EO features with the extraction conditions, were obtained and used to identify the best experimental conditions able to maximize the yield and thymol concentration. The optimized MAE procedure assures an EO with a higher yield and thymol amount compared with the standard hydrodistillation procedure.

## 1. Introduction

The annual plant *Trachyspermum ammi* L. Sprague (syn. *Carum copticum* Benth. and Hook. f.), belonging to the family of Apiaceae and known with different local names such as ajowan or ajwain [1], grows and is cultivated in several regions of Eastern India, Iran, Iraq, Pakistan, Egypt, Afghanistan and China [2,3]. Ajowan is used for fruits (schizocarps, commonly named “seeds”) which are easily accessible and inexpensive; indeed, the price in Iran is about 1.5 USD/kg. The beneficial effects and pharmacological properties ascribed to these fruits are known from centuries and largely exploited in Middle Eastern traditional medicine [4]. In fact, oral administration is effective in treating neurological pathologies such as tremors and paralysis, and also in case of gastro-intestinal disorders including nausea, vomiting, reflux, abdominal colic, liver disease, splenic pain and loss of appetite [5]. Moreover, ajowan fruits are used in the case of respiratory affections, namely cough, bronchitis, pneumonia and dysphonia. They are also included in topical preparations, often with honey or egg white, exerting analgesic and anti-inflammatory effects [6]. The ministerial guidelines recommend its application to improve respiratory and digestive functions. Ajowan fruits essential oil (EO) contains bioactive compounds, among which thymol is predominant. This EO is endowed with significant insecticidal activity against mosquitoes [7,8,9], termites [10], cockroaches [11] and other pests [12,13,14]. As an acaricide, ajowan EO is effective, in particular, against Dermanyssus gallinae De Geer [15]. This EO also exhibits antimicrobial properties against several pathogens [16,17,18,19,20,21,22] and also antimycotic effects [23,24]. A recent study demonstrated important antioxidant properties and cytotoxic effects on colon carcinoma cells for the ajowan EO [25]. All the aforementioned biological activities can be attributed to the EO major chemical constituents, among which thymol is usually the predominant compound. Thymol possesses several interesting applications, with relevant practical and commercial value [26]. Due to its antimicrobial and antiseptic properties, thymol is included in some products for body and mouth care [27,28]. In particular, thymol is used in case of caries, throat and gingivitis infections; indeed, thymol is one of the ingredients of Listerine^®^ mouthwash (thymol 0.064%) [29] and Cervitec^®^ Plus protective varnish (thymol 1%) [30]. Thymol, being classified as a GRAS (generally recognized as safe) substance by the FDA [31], possesses several interesting applications, with relevant practical and commercial value [26]. This compound is an ideal component of biodegradable packages to reduce the proliferation of bacteria and fungi, improving the food shelf life [32,33,34]. As an acaricidal agent, thymol shows efficacy against several mites [35,36,37] and is incorporated in the gel formulation Apiguard^®^ (thymol 25%) to control the parasite Varroa destructor Anderson and Trueman which threatens honeybees [26,38]. The insecticidal activity of thymol was confirmed especially against mosquitoes [39,40]. Consequently, ajowan EO may be applied in the preparation of safe and eco-friendly formulations to be used as an alternative to the synthetic products.

Given the economic value of ajowan and its increasing demand across the globe, the extraction method used for its EO is of paramount importance in terms of quality and quantity of the final product. Currently, most of the studies reported on the T. ammi EO rely on conventional extraction techniques such as hydrodistillation. However, in recent years novel extraction techniques have spread due to the need for more effective and environmentally friendly alternatives [41]. Among them, microwave-assisted extraction (MAE) is promising due to its high extraction efficiency, together with other advantages in terms of energy consumption, process length and environmental impact. In addition, the MAE process is characterized by a high versatility and can be tuned to operate on several parameters [42,43]. Microwave power, extraction time, presence of water, water-to-plant matrix ratio, the number of extraction cycles as well as biomass pre-treatments (e.g., moistening, drying, milling etc.) represent operative parameters commonly applied or adjusted during the MAE, even if in several cases their relevance or usefulness is still not well-understood or demonstrated.

The aim of this work was to study for the first time, the effect of different MAE parameters on the quality and quantity of ajowan EO, and afterwards, to optimize the process in terms of yield and thymol content. The design of experiment (DoE) approach represents a convenient methodology to study and optimize a process where several factors can be effective. In the case of ajowan fruits, seven factors were identified as potentially relevant: microwave power (MP), extraction time (ET), water-to-seed ratio (WSR), preliminary moistening time (PMT), preliminary milling process (PMP) and extraction cycles (EC). Due to the high number of factors to be investigated, the application of a single experimental plan would result in a huge number of experiments (a central composite design for 6 factors requires over 50 runs) and consequently an excessive cost and unfavourable cost/benefit ratio. For this reason, it was decided to carry out a two-step DoE, where the first step is represented by a screening design and the second one by a response surface methodology (RSM) design. The screening’s aim was to identify the factors that may influence the studied process, while the RSM allowed the building of models describing the relationships between factors and responses, and provided a prediction tool of the MAE performances.

## 2. Results and Discussion

### 2.1. Screening of the MAE Parameters

The DoE approach is a convenient and efficient methodology to study industrial processes, defining the relationship between factors and responses and optimizing the best experimental conditions. However, when the factors to be investigated become numerically consistent, adopting a single DoE is not particularly efficient due to the high number of experiments required and the consequently high cost and time-consuming experimental plan. For this reason, it was decided to carry out a preliminary screening design aimed to identify the most relevant factors. For the same reasons, in this stage the EO composition was not determined by GC-FID analysis, but rather estimated using a less expensive and time-consuming procedure such as the determination of the density and refractive index. According to Delgado-Ospina et al., the density can be considered a qualitative indicator while the refractive index may even be used to accurately determine the content of thymol in EOs [44].

**Figure 1:**
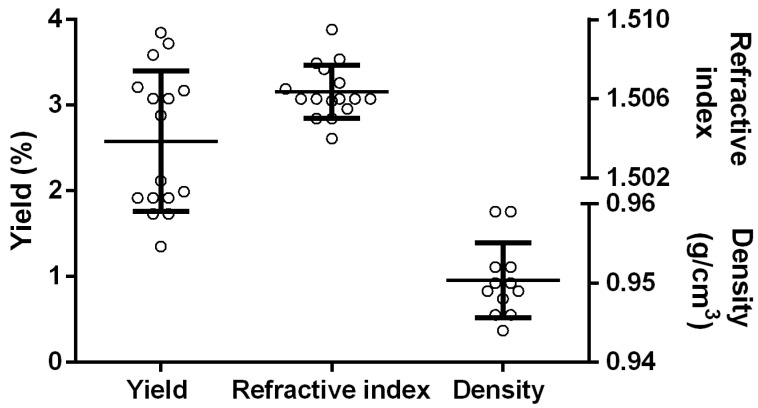
Values of yield, refractive index and density of all the essential oils extracted during each single run of the fractional factorial design.

Before performing the FFD analysis, the responses determined for each of the 16 experimental runs were preliminarily examined using individual value plots. The graph (Figure 1) shows that the yield is characterized by a great variation within all the experimental runs, which is different from the density and refractive index values. The range of ajowan EO yields was between 1.6 and 3.7% (*v*/*w*), which is within the ranges reported in the literature [25,45,46]. The measured values of the refractive index and density differ at the third decimal digit, that is the lower sensibility limit of the used instruments; hence, they cannot be considered reliable to discriminate the thymol concentration. For this reason, the screening step was performed only to study one response, i.e., the yield.

**Figure 2:**
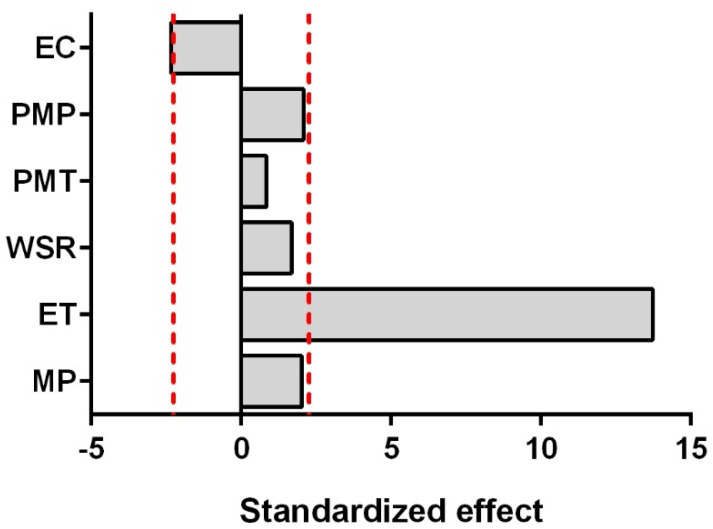
Pareto plots showing the factors’ influence on the yield of essential oils determined in the fractional factorial design. The red line represents the statistically significant limit when the variables are reported in terms of standardized effect (*t*-value of the coefficient). EC: extraction cycles; PMP: preliminary milling process; PMT: preliminary moistening time; WSR: water-to-seed ratio; ET: extraction time; MP: microwave power.

The selected linear model fits very well the yield values with an *R*^2^*_adj_* of 0.930 and an *R*^2^*_pred_* of 0.868 (all the details of ANOVA, model and coefficient analysis results are reported in the Table S1 and Figure S1 of the supplementary information file). The general results of the screening analysis are reported using a Pareto plot (Figure 2). The ET is by far the most relevant parameter characterized by a positive effect. The EC represented the only other significant effect. Specifically, the yield was higher when a single EC was performed. Such results can appear anomalous compared with the literature [42,43]. However, it is fundamental to take into account how the cycles are compared. In this study, the duration of each EC was set in order to keep the total ET constant. In the literature, the results refer to processes characterized by different numbers of cycles but also different ET. Consequently, the results of this study indicated that the higher yield found in the literature for multi-cycle extraction processes is likely due to an increase in the ET rather than to an effect of the cycles themselves. Such a result is valid within the conditions herein applied. Nevertheless, we need to point out that an exact comparison with literature data is difficult due to the different vegetable matrices studied and to the specific experimental conditions applied.

None of the other investigated parameters affected the extraction yield in a statistically significant manner. The result of MP is rather surprising, since it usually represents a relevant parameter in the MAE processes [47,48,49]. However, it is important to highlight that the *p*-value was close to the limit (0.068) and the investigated range was not particularly wide.

### 2.2. Central Composite Design (CCD)

The CCD was applied to identify suitable mathematical models able to explain the relationships between the variables and responses and to provide predictions about how the responses vary as a function of the factors. From the screening analysis, the factors WSR, PMT and PMP did not affect the yield of the process, thus they were kept constant in CCD. In addition, the EC was kept constant at the most favourable values (a single cycle). This choice was due to the nature of the parameter, extraction cycles. In fact, this factor represents a discrete numerical parameter (categorical factor) and consequently it can be only set to specific values within its experimental domain. For the MP the choice was more complex. The MP did not result as statistically significant during the screening step, although the *p*-value was close to the limit. Moreover, the highest value tested (1.2 W/g) in the FFD was quite far from the maximum limit of the instrument (1800 W, that for an amount of biomass including water of 1.3 kg corresponds to a MP of 1.37 W/g). For these reasons, it was decided to select the MP for the CCD analysis; however, its experimental domain was shifted up to the instrumental limit. The ET was also included in the CCD. However, in this case it was decided to shift its domain toward higher values in order to assure a likely better yield according to the FFD results. The comparison between the CCD and FFD experimental domain is reported in Figure S2 in the supplementary information file.

**Figure 3:**
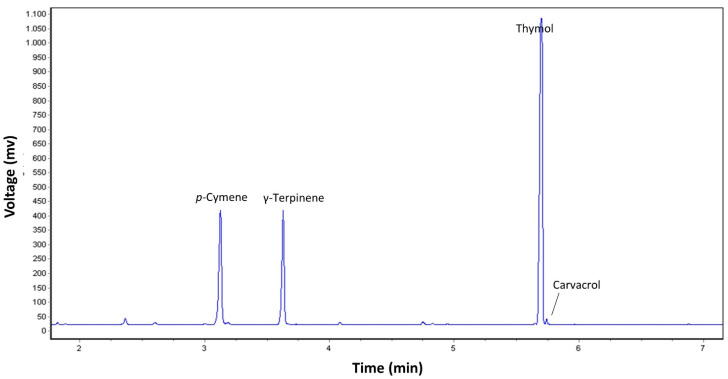
Chromatograms obtained by gas chromatography coupled with flame ionization detection of the ajowan essential oil obtained by microwave-assisted extraction (the profile refers to run n° 10 of the central composite design).

In addition to the yield, in the CCD study the analysis of EO chemical composition was included. The chemical profile of the ajowan EO was mostly dominated by three constituents, namely thymol, p-cymene and γ-terpinene, accounting for more than 90% of the total composition (Figure 3). In the EOs extracted during the experiments of the CCD study, thymol was by far the most abundant one, with percentage values ranging from 63% to 73%; p-cymene (13–16%) and γ-terpinene (10–14%) accounted for 23–30% of the total composition. Their presence can be justified by their pivotal role in the biosynthesis of thymol [50]. Carvacrol, which is biosynthetically related to thymol, in all cases was found in low percentages (<0.5%). The EO profiles obtained by MAE are consistent with those reported in most of the literature reports on ajowan EOs obtained by conventional hydrodistillation [7,25,46]. To the best of our knowledge, there is only one study on the composition of the ajowan EO obtained by MAE [51]. The authors found an overlapping profile with those found in our study, with thymol (60.3%), p-cymene (21.2%) and γ-terpinene (16.4%) as the predominant compounds.

The EOs yield and the concentration of the main three components plus carvacrol were analysed using the CCD. The best models for each response identified during the CCD analysis are reported in Table 1. The models fit in a satisfactory manner, the experimental data for all the responses, with the exception of the carvacrol amount in the EO, showing a significant regression without suffering from lack of fit. In addition, the issue of multicollinearity and the violation of the regression assumptions can be excluded, as shown by the coefficient and residual analysis, respectively (Table S2 and Figure S3 in the supplementary information file). Concerning carvacrol, the model is completely inadequate: the regression is not significant and the values of *R^2^_adj_* and *R^2^_pred_* are very low. Such a result is likely due to its intrinsic variability, since the lack of fit does not result as statistically significant. Possibly, the cause is the very low carvacrol concentration in EOs. In this situation, even small errors during its quantification can have a huge impact on the general variability.

**Figure 4:**
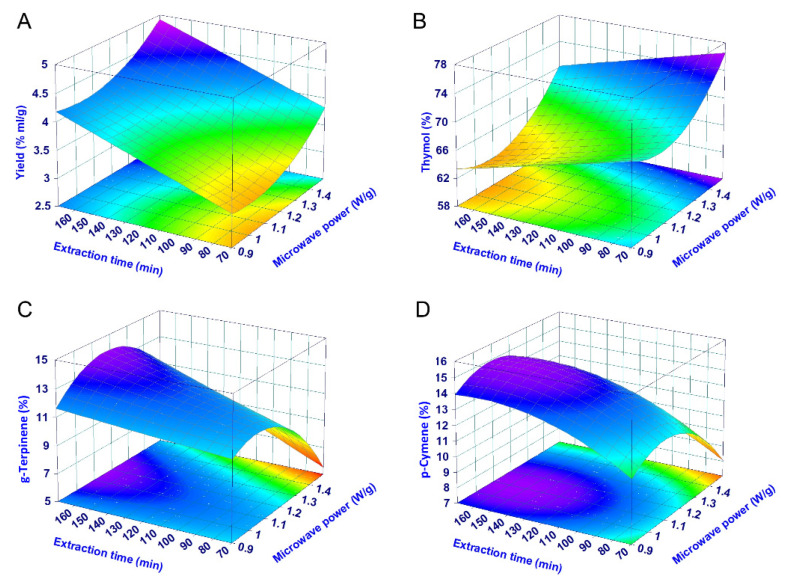
Surface plots showing the effect of the irradiation power and extraction time on the (**A**) yield, (**B**) thymol, (**C**) γ-terpinene and (**D**) p-cymene concentration in the essential oil.

The relationships between the responses and variables can be visualized using surface plots (Figure 4). Interestingly, the two evaluated variables (MP and ET) affect the four responses in a different way. The highest EO yields require the application of high MP and ET values, while the highest thymol concentrations are achieved operating at high MP and lower ET values. The p-cymene and γ-terpinene concentrations are instead affected in a similar manner by the extraction parameters; the conditions favouring their highest recovery appear to be opposite with respect to those enhancing the content of thymol. Thus, it should be theoretically possible to obtain an ajowan EO enriched in thymol or in p-cymene and γ-terpinene by tuning the above extraction parameters. On the other hand, the highest EO yields require intermediate conditions with respect to those favouring the single components (Figure 4). This situation is also confirmed by evaluating the Pearson correlation between all the responses. In fact, none of the responses representing the concentration of the bioactive markers is correlated with the yield, while p-cymene and γ-terpinene concentrations are positively correlated with one another and negatively correlated with the thymol concentration (Figure S4 in the supplementary information file).

### 2.3. MAE Optimization and Model Validation

**Figure 5:**
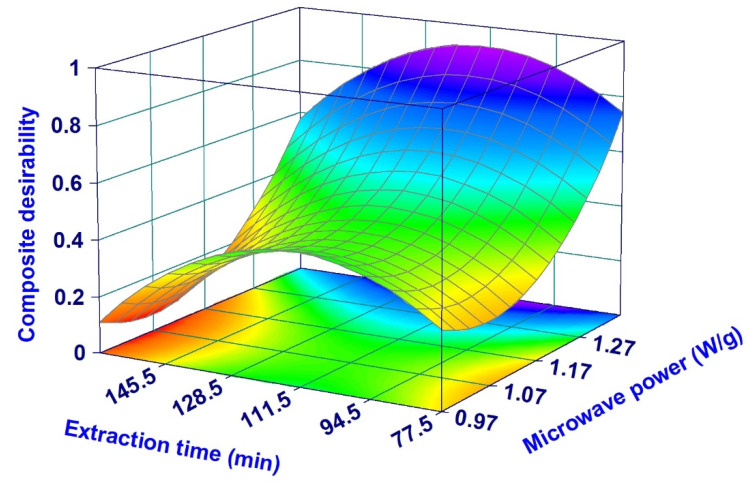
Surface plots of the effect of the irradiation power and extraction time on the composite desirability satisfying the criteria of yield and thymol content maximization.

For the optimization of the extraction conditions, it was decided to focus on the EO yield and thymol content since they are markers of the ajowan economic value. These were both optimized using the desirability procedure aimed at maximizing their values. The composite desirability function is reported in Figure 5 using a surface plot. As expected from the shape of the single surface plots of the two responses (Figure 4), the optimal conditions required to maximize the yield and thymol concentration are represented by high MP and intermediate ET values. Interestingly, it seems that a second peak (a local maximum) can be located in the desirability surface at the same ET but with lower MP (at around 1 W/g). However, in this case it is necessary to highlight that the highest composite desirability of this second peak is only around 0.4, a value too low for any practical application.

From the composite desirability function the best experimental conditions able to maximize yield and thymol concentration were identified (E_OPT_). In addition to the E_OPT_, the desirability procedure was applied to define the other two sets of conditions, i.e., E_V1_ and E_V2_, which are necessary to validate the models. E_V1_ aims to maximize only the yield, while E_V2_ maximizes the yield and minimizes the concentration of thymol. The experimental conditions of E_OPT_, E_V1_ and E_V2_, together with the composite desirability, the predicted means and 95% intervals of predictions are reported in Table 2.

**Figure 6:**
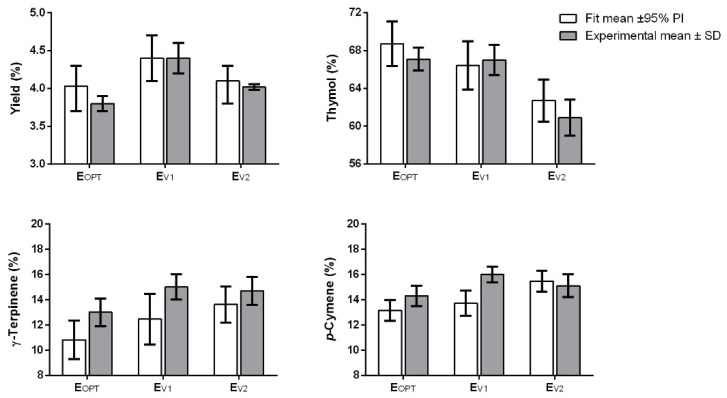
Comparison between the models’ predictions (fit mean and 95% of interval of prediction) and the experimental results of the runs used for the model validation.

The comparison between the predicted values and those experimentally obtained for all the considered responses are reported in the Figure 6. Interestingly, the models’ predictions are accurate in the case of yield and thymol, with the experimental mean values always within the 95% interval of prediction. For γ-terpinene and p-cymene the values predicted by the models were accurate only for the condition E_V2_. However, it is necessary to point out that despite the lack of accuracy, the differences between the model predictions and the experimental values are in the range of 1–2%. For example, in the worst case (e.g., concentration of p-cymene in the E_V1_), the difference between the experimental mean value and the highest limit of the 95% interval of prediction is 1.3%. Thus, the low accuracy does not represent a problem from a practical point of view and the models can be used to optimize the content of γ-terpinene and p-cymene. Comparing our data with those reported by Lucchesi et al. [51] we notice that the optimization allows us to obtain a greater value of the yield and thymol content. The lower irradiation capacity of the extractor (maximum delivered power of 1000 W vs. 1800 W) and extraction time (60 min vs. 126 min) used by Lucchesi et al. can explain such differences.

### 2.4. Comparison of the EO Yield and Chemical Compositions Obtained by MAE and HD

**Figure 7:**
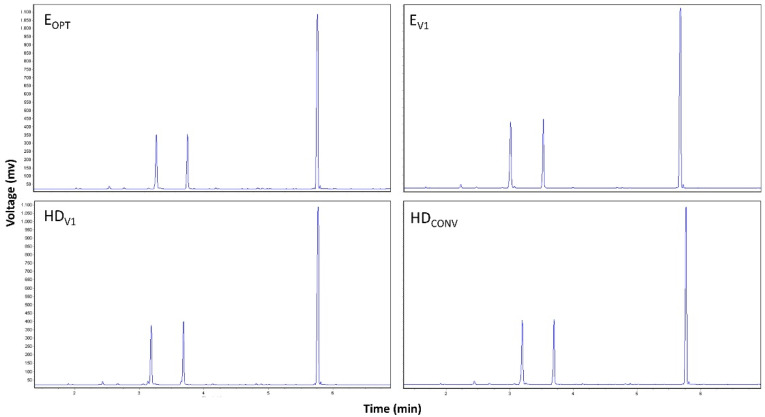
GC–FID chromatograms of the ajowan essential oil obtained by microwave-assisted extraction (E_OPT_, E_V1_) and hydrodistillation (HD_V1_ and HD_CONV_).

**Figure 8:**
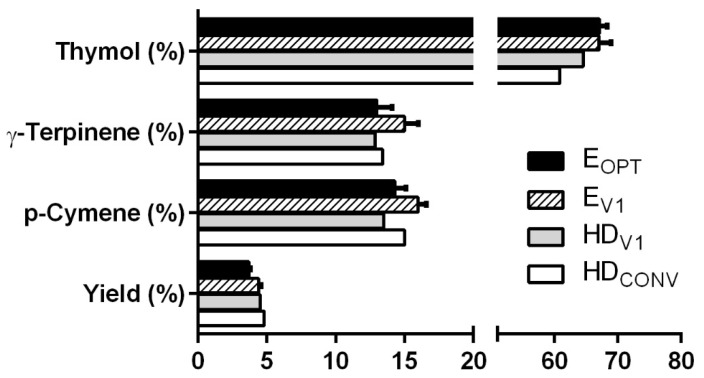
Comparison between yield and the thymol, γ-terpinene and p-cymene concentration in the essential oil obtained by hydrodistillation (HD_V1_ and HD_CONV_) and microwave-assisted extraction (E_OPT_, E_V1_).

The chemical compositions obtained for ajowan EO extracted by MAE and HD are depicted in Figure 7 whereas the complete compositions are reported in Table S3 of the supplementary information file. From a qualitative point of view, all the chromatograms were overlapping. On the other hand, the optimized MAE conditions allowed for the obtaining of higher amounts of thymol than HD (67% vs. 60–64%, respectively), while the levels of p-cymene and γ-terpinene were quite similar (Figure 8). Interestingly, MAE and HD extractions conducted with the same duration provided the same yield (around 4.5%), while HDCONV carried out for 4 h assured the highest yield (4.8%). This result appears consistent with the FFD and CCD analysis outputs, where ET is the most important parameter. Thus, we assume that the most important parameters to optimize during MAE are likely the same for HD, taking into account the intrinsic differences between the two extraction techniques.

## 3. Materials and Methods

### 3.1. Plant Material

Schizocarps of T. ammi fruits were collected at the ripening stage, from wild plants growing in Ardabil, Ardabil Province, Iran (38°18′ N; 48°19′ E; 1346 m a.s.l.), in August 2019. The voucher specimen was identified and deposited under the codex TAM 4512, in the Herbarium of the University of Maragheh, Maragheh, Iran.

### 3.2. Sample Pre-Treatment

Based on the extraction experimental conditions described below (Section 2.4), dry fruits of T. ammi were used as received or subjected to grinding and/or moistening. A plant grinder from Albrigi Luigi Srl, Verona, Italy (code E0585), with a power of 1100 W, was employed to reduce the biomass into <1.5 mm particles and this process was repeated twice. The effect of the preliminary milling process on the fruits’ particle size is detail reported in the supplementary information file (Figure S5). The moistening treatment was performed for 4 h prior to extraction in distilled water, as described in Section 2.3, with a precise matrix-to-solvent ratio.

### 3.3. Microwave-Assisted Extraction (MAE)

For this study, a Milestone ETHOS X (Milestone, Sorisole, Italy) device was employed, namely a multimode microwave reactor of 2.45 GHz, with 1800 W maximum power (950 W delivered by each of the two magnetrons) and an infrared sensor to control the temperature. The extraction runs were performed loading 1300 g of plant material including added water in a 5 L Pyrex glass reactor with a glass cover and at atmospheric pressure using a steel Clevenger-type apparatus (fragrances set up). This system was coupled to a Chiller Smart H150-2100S (Labtech srl, Sorisole, Bergamo, Italy) that maintained the temperature of the recirculating water at 8 °C. Distillation occurred when the temperature shown by the infrared sensor was higher than 90 °C. The experimental conditions of each extraction run were performed by varying the microwave power (MP), extraction time (ET), water-to-seed ratio (WSR), preliminary moistening time (PMT), preliminary milling process (PMP) and extraction cycles (EC), as planned by the DoE (Section 3.4). The parameter WSR defined the percentage of the fruit amount with respect to the total weight of the sample (fruits plus water) loaded in the reactor. As an example, a WSR of 4 indicates that the sample loaded in the reactor was constituted by 52 g of fruits and 1248 g of water (the weight of samples loaded was always kept constant at 1300 g). PMT specifies whether the water was added to the plant material 4 h prior to or immediately before the extraction. EC refers to the number of cycles used, i.e., 1, meaning that the extraction was carried out by continued microwave irradiation; and 2, meaning that the whole process was split in two irradiation steps separated by the time necessary to lower the temperature in the reactor to 50 °C. Indeed, the whole irradiation period of each run depends only on the extraction time and not the number of cycles. The obtained EOs were kept at 4 °C inside glass vials closed with PTFE-silicon septa (Sigma-Aldrich, Milan, Italy) until analysed.

### 3.4. Design of Experiment (DoE)

#### 3.4.1. Screening Design

The identification of the parameters potentially affecting the MAE process was performed using a two-level quarter fractional factorial design (FFD), which is defined by:

(1) $$N=l^{f-\frac{p}{2}}$$

where *N* is the number of experimental runs, *l* is the number of levels (2 in this case), *f* the number of factors (6 in this case) and *p* is the partitioning of the design. In this case, it was decided to fraction the original factorial design to a quarter, so the value of *p* is 4. The FFD selected requires 16 experimental runs which are determined using the generators G1 = 234 and G2 = 1234 and is characterized by a resolution of IV [52]. The resolution describes how much the effects in an FFD are aliased with other effects, which is the ability of the design to allow an independent estimation of the main effects and their interactions. A resolution of IV represents a good compromise for a screening design since it allows an independent estimation of the main effects, while two-factor interactions are aliased between them [52]. The complete list of all the 16 extraction runs with the corresponding coded and uncoded variables is reported in Table 3. Each extraction run was characterized in terms of:

- EO yield (%), calculated as follows:
  (2) $$EO\;yield\;\left(\%\right)=\frac{weight\;of\;EO\;\left(g\right)}{weight\;of\;dry\;biomass\;\left(g\right)}\times 100$$
- EO density (g/cm^3^).
  Determined using an oscillating U-tube density meter (DA-100M, Mettler Toledo, Greifensee, Switzerland) at 20 °C;
- EO refractive index.

Determined with an Abbe refractometer (NAR-1T LIQUID, Atago Co. ltd., Tokyo, Japan) at 20 °C.

For each of the three responses, all the results of the 16 runs were analysed by multilinear regression using a liner model (suitable for a resolution IV design):

(3) $$y=\beta_{0}+\sum_{i=1}^{n}\beta_{i}\text{·}x_{i}$$

where *y* is the response, *β*_0_ is the model constant, and *βi* is the coefficient corresponding to the variables *x_i_* (linear terms).

The fitting procedure of the experimental results with the linear model was then evaluated through the analysis of variance (ANOVA) coefficient and residual analyses. The screening design and analysis were performed with the Minitab 18 statistical software.

#### 3.4.2. Response Surface Methodology (RSM) Design

The RSM allows the building of accurate analytical models able to explain how the independent variables (factors) affect the responses. The RSM design was performed on the relevant continuous numerical factors identified after the screening design, namely MP and ET. The un-relevant numerical factors as well as all the categorical ones were kept constant (12 for the WSR, 1 for EC, while milling and moistening were not performed).

The effect of MP and ET was analysed using a two-factors central composite design (CCD), composed by four (2^2^) factorial experiments, four (2 × 2) axial experiments and three central experiments. The presence of the axial points and three replicates of the central points, assures the design rotatability and a uniform precision within the experimental domain [53]. The full list of the experimental run is reported in Table 4.

Each extraction run was characterized in terms of:

- EO yield (%), calculated as in the previous section;
- Concentration of the EO marker compounds, namely thymol, p-cymene, γ-terpinene and carvacrol (g/100 g of EO), determined by GC-FID as reported in the Section 3.6.

Each single response was then analysed by multilinear regression using a full quadratic model:

(4) $$y=\beta_{0}+\overset{n}{\underset{i=1}{\sum }}\beta_{i}\text{·}x_{i}+\;\overset{n}{\underset{i=1}{\sum }}\beta_{ii}\text{·}x_{i}^{2}+\overset{n}{\underset{i<j}{\sum }}\beta_{ij}\text{·}x_{i}x_{j}$$

where *y* is the response, *β*_0_ is the model constant, *β_i_* is the coefficient corresponding to the variables *x_i_* (linear terms), *β_ii_* are the coefficients associated with the variables *x_ii_* (quadratic term) and *β_ij_* are the coefficients associated with the variables *x_i_x_j_* (first-order interaction terms).

The obtained full quadratic models were subjected to a reduction procedure in order to improve the precision of the estimated coefficients of the retained variables, to minimize the mean square error and, in general, to satisfy the principle of parsimony [54,55]. The model’s reduction was performed by stepwise regression in backward elimination mode, identifying the most suitable models by evaluating the adjusted coefficient of multiple determination (*R*^2^*ad*j), the predicted coefficient of multiple determination (*R*^2^*pred*) and the Mallows’ Cp statistic [47]. The final models were evaluated through ANOVA, coefficient and residual analyses.

Finally, a multiple responses optimization procedure was carried out using the desirability method to define the more suitable experimental conditions able to provide satisfactory results for the yield and thymol content [56]. For both the responses, a linear partial desirability function that maximizes both responses was chosen [57], setting the target values and the unacceptable limits as a function of the possible results obtainable within the experimental domain. This extraction run is reported in the manuscript as the optimized extraction (E_OPT_).

In addition to the extraction procedure able to maximize the yield and thymol concentration, two further sets of experimental conditions were identified using the desirability method:

Extraction V1 (E_V1_), selected to assure the maximum yield independently by the main component concentrations.

Extraction V2 (E_V2_), selected to assure the maximum yield and the lowest concentration of thymol.

E_OPT_, E_V1_ and E_V2_ were performed in triplicate and their EOs characterized in terms of yield and concentration of the main compounds (Section 3.7). The model validation was performed by comparing the features of the EOs of E_OPT_, E_V1_ and E_V2_ with those predicted by the models (predicted fit values and 95% prediction interval) [58].

The CCD design, the model fitting, reduction and analysis, as well as multiple responses optimization and the calculus of the 95% prediction intervals of the predicted value were carried out using the software Minitab 18.

### 3.5. GC-MS Analysis

A total of 6 µL of the ajowan EOs and 594 µL of analytical-grade n-hexane (Sigma-Aldrich, Milan, Italy) were injected in split-mode (split ratio 1:50) into an Agilent 6890 N GC-MS system, endowed with a 5973 N single quadrupole detector and an autosampler 7863. As a stationary phase, a 5% phenyl-methylpolysiloxane coated capillary column (Agilent HP-5MS, 30 m length, 0.25 mm internal diameter, 0.1 μm film thickness) was used. The temperature program was set as follows: 5 min isothermal at 60 °C, then ramp at 4 °C/min to 22 °C and, finally, ramp at 11 °C/min until 280 °C, maintained for 15 min. The flow rate of the carrier gas (helium 99.5%) was 1 mL/min and both the injector and detector were at 280 °C. The mass spectra were achieved between 29.0 uma and 400.0 uma in a full scan through the electron impact mode (EI, 70 eV). The major chemical constituents of the T. ammi EOs, namely p-cymene, γ-terpinene, and thymol, as well as carvacrol, were identified using analytical standards (Sigma-Aldrich, Milan, Italy), whereas the other components were identified by combining the temperature-programmed retention indices (RIs) and the mass spectra (MS), as detailed in our previous study [59].

### 3.6. Thymol Isolation and Identification through NMR Analysis

In some ajowan EO samples, the formation of a white crystalline precipitate was observed; thus, we decided to isolate it. Firstly, we filtered it and washed it several times with cold hexane (−20 °C) using a Gooch funnel under a vacuum. Then, the solid product was dried, dissolved in deuterated DMSO and then subjected to 1H NMR analysis by a VARIAN Mercury Plus 400 MHz spectrometer using TMS (tetramethylsilane) as internal standard. The chemical shift values were indicated as δ values (ppm) and coupling constants (J) were reported in hertz. Proton chemical data were as follows: chemical shift, multiplicity (singlet (s), doublet (d), doublet of doublets (dd), triplet (t), doublet of triplets (td), quartet (q), multiplet (m), broad singlet (brs)) coupling constant (s), and integration. The compound was identified as pure thymol: 1H NMR (DMSO-d6): 1.09 (s, 3H, CH3); 1.11 (s, 3H, CH3); 2.14 (s, 3H, CH3); 3.12 (m, 1H, CH(CH3)2); 6.52 (d, 1H, Ar, J = 7.21 Hz); 6.56 (s, 1H, Ar); 6.93 (d, 1H, Ar, J = 8 Hz); 9.05 (brs, 1H, OH). The 1H NMR spectrum and the image of the isolated crystals are reported in the supplementary information file (Figure S6).

### 3.7. Quantification of The Bioactive Markers by Gas Chromatography Coupled with Flame Ionization Detection (GC-FID)

The quantification of p-cymene, γ-terpinene, thymol and carvacrol in ajowan EOs was carried out by using a gas chromatograph coupled with flame ionization detection (GC-FID, 6850, Agilent Technologies, Santa Clara, CA, USA). Analytical standards of the above compounds were purchased from Sigma-Aldrich. A 20 μL aliquot of ajowan EO was diluted to a final volume of 2 mL with an analytical grade diethyl ether. The injection of the homogenized solution was performed in split mode (split ratio 1:30) and the injection volume was 0.5 μL. The injector temperature was 300 °C. The carrier gas was hydrogen produced by a generator PGH2−250 (DBS Analytical Instruments, Vigonza, Italy). The initial gas flow in the column was 3.7 mL/min. Chromatography was performed on an HP-5 capillary column (5% phenyl-methylpolysiloxane, 30 m length, 0.32 mm internal diameter, 0.25 μm film thickness, Agilent Technologies, Santa Clara, CA, USA). The oven temperature was held at 60 °C for 3 min, then raised until 350 °C at 25 °C/min and held for 1 min, for a total run time of 15.60 min. The FID temperature was set at 360 °C, and hydrogen and air flow were 40 mL/min and 400 mL/min, respectively. The quantification was performed by using the calibration curves obtained for p-cymene, γ-terpinene, thymol and carvacrol. The curves were obtained by stock standard solutions at 7 different concentrations in the range 0.05–5 mg/mL for p-cymene and γ-terpinene, 0.15–15 mg/mL for thymol and 0.005–0.15 mg/mL for carvacrol. Determination coefficients ranged from 0.9989 to 0.9995.

### 3.8. Hydrodistillation (HD)

For comparative purposes, two different HDs were performed and compared with the MAE. In the first one, named HDV1, the same length of the MAE validation run E_V1_ (162.4 min) was applied, while for the second one, named HDCONV, a conventional distillation time of 240 min was set. In general, ajowan dry fruits were placed into a 6 L round flask, heated by a mantle system Falc MA (Falc Instruments, Treviglio, Italy) coupled with a glass Clevenger-type apparatus using the same weight and WSR of those used in MAE. The distilled EOs were decanted, separated from the aqueous layer and maintained in the fridge inside glass vials with PTFE/silicon caps until chemical analyses.

## 4. Conclusions

In this study we used for the first time a DoE approach to screen the operative conditions during the ajowan EO microwave-assisted extraction and to model and optimize the EO yield and chemical profile. The extraction time revealed to be the most important factor affecting the yield and composition followed by the microwave power. The thymol content was maximized by short extraction time (80 min) and high irradiation power (1.37 W/g), while the content of the other minor components (p-cymene and γ-terpinene) were negatively correlated with the above parameters. Yield is instead favoured by intermediate conditions, that are long extraction times (160 min) and high irradiation power (1.37 W/g) with respect to those maximizing thymol or p-cymene and γ-terpinene. The mathematical models were accurate in the prediction of yield and thymol content allowing the optimization of both responses at the same time. Compared with conventional hydrodistillation operating at the same extraction time, the microwave-assisted extraction assured higher amounts of thymol and comparable yields. Interestingly, the extraction time influences hydrodistillation in a similar way to MAE. Although hydrodistillation can provide comparable results, the microwave-assisted extraction has the great advantage of versatility since its parameters can be easily and precisely adjusted.

## Figures and Tables

**Table 1:** Best mathematical model for each response and its evaluation parameters: coefficients of determinations (*R*^2^*_adj_* and *R*^2^*_pred_*). Mallows’ Cp statistic and ANOVA results (*p*-values of regression and lack of fit).

Response	Best Model ^a^	$R^{2}$	$R_{adj}^{2}$	$R_{pred}^{2}$	Mallow’s Cp	*p*-Value Regr ^b^	*p*-Value Lof ^b^
Yield (%)	Y = 5.92 − 7.15MP + 0.011ET + 3.49MP^2^	0.940	0.914	0.813	2.83	***	ns
Thymol (g/100 g)	Y = 126.3 − 99.7MP − 0.063ET + 46.3MP^2^	0.914	0.878	0.807	2.43	***	ns
γ-Terpinene (g/100 g)	Y = −16.1 + 54.7MP − 0.058ET − 28.55MP^2^ + 0.075MP * ET	0.880	0.799	0.675	4.09	**	ns
p-Cymene (g/100 g)	Y = −25.19 + 55.3MP + 0.125ET − 25.12MP^2^ − 0.0004ET^2^	0.965	0.942	0.862	4.39	***	ns
Carvacrol (g/100 g)	Y = −0.143 + 0.536MP + 0.004ET − 0.004MP * ET	0.387	0.125	<0.001	2.14	ns	ns

^a^ The models are reported using the coefficients calculated from the uncoded variables. ^b^ The results of *p*-value columns are reported as follows: ns = *p* > 0.05; * 0.05 < *p* < 0.01; ** 0.01 < *p* < 0.001; *** *p* < 0.001.

**Table 2:** MAE experimental conditions. Desirability values and type. Predicted values and the 95% interval of predictions of the optimized run (EOPT) and of the two validation runs (E_V1_ and E_V2_) for the all the responses.

Extraction	MAE Conditions		Composite Desirability	Responses Optimized with Desirability	Desirability Function	Other Responses	Predicted Value	95% Interval of Prediction
	Power (W/g)	Time (min)						
E_OPT_	1.37	126.4	0.88	Yield	Maximize		4.0	3.7–4.3
				Thymol	Maximize		68.7	66.3–71.1
						γ-terpinene	10.8	9.3–12.4
						p-cymene	13.2	12.3–14.0
E_V1_	1.37	162.4	1	Yield	Maximize		4.4	4.1–4.7
						Thymol	66.4	63.9–69.0
						γ-terpinene	12.5	10.5–14.5
						p-cymene	13.7	12.7–14.7
E_V2_	1.17	162.4	0.98	Yield	Maximize		4.1	3.8–4.3
				Thymol	Minimize		62.7	60.5–64.9
						γ-terpinene	13.6	12.2–15.1
						p-cymene	15.5	14.6–16.3

**Table 3:** Experimental conditions both in uncoded and coded variables of the sixteen runs carried out according to the screening design.

Run	Uncoded Variables ^a^						Coded Variables ^a^					
	MP (W/g)	ET (min)	WSR (%)	PMT (h)	PMP	EC (No)	MP	ET	WSR	PMT	PMP	EC
1	0.8	50	4	0	N	1	−1	−1	−1	−1	−1	−1
2	1.2	50	4	0	Y	1	+1	−1	−1	−1	+1	−1
3	0.8	120	4	0	Y	2	−1	+1	−1	−1	+1	+1
4	1.2	120	4	0	N	2	+1	+1	−1	−1	−1	+1
5	0.8	50	12	0	Y	2	−1	−1	+1	−1	+1	+1
6	1.2	50	12	0	N	2	+1	−1	+1	−1	−1	+1
7	0.8	120	12	0	N	1	−1	+1	+1	−1	−1	−1
8	1.2	120	12	0	Y	1	+1	+1	+1	−1	+1	−1
9	0.8	50	4	4	N	2	−1	−1	−1	+1	−1	+1
10	1.2	50	4	4	Y	2	+1	−1	−1	+1	+1	+1
11	0.8	120	4	4	Y	1	−1	+1	−1	+1	+1	−1
12	1.2	120	4	4	N	1	+1	+1	−1	+1	−1	−1
13	0.8	50	12	4	Y	1	−1	−1	+1	+1	+1	−1
14	1.2	50	12	4	N	1	+1	−1	+1	+1	−1	−1
15	0.8	120	12	4	N	2	−1	+1	+1	+1	−1	+1
16	1.2	120	12	4	Y	2	+1	+1	+1	+1	+1	+1

^a^ Abbreviation for coded and uncoded variables are: MP: microwave power; ET: extraction time; WSR: water-to-seed ratio; PMT: preliminary moistening time; PMP: preliminary milling process; EC: extraction cycle.

**Table 4:** Experimental conditions both in uncoded and coded variables of the eleven runs carried out according to the central composite design (CCD).

Run	Point Type ^a^	Uncoded Variables ^b^		Coded Variables ^b^	
		ET (min)	MP (W/g)	ET	MP
1	F	90	1	−1	−1
2	F	90	1.31	−1	1
3	F	150	1	1	−1
4	F	150	1.31	1	1
5	A	120	0.94	0	−1.41421
6	A	120	1.37	0	1.41421
7	A	78	1.155	−1.41421	0
8	A	162	1.155	1.41421	0
9	C	120	1.155	0	0
10	C	120	1.155	0	0
11	C	120	1.155	0	0

^a^ This point type column defines whether a certain set of experimental conditions represents a factorial (F). axial (A) or central (C) point in the CCD experimental domain. ^b^ Abbreviations for coded and uncoded variables are: MP: microwave power; ET: extraction time.

## Data Availability

Data is contained within the article and supplementary material.

## Supplementary Materials

The following are available online at https://www.mdpi.com/article/10.3390/ph14080816/s1, Figure S1: FFR Residuals analysis of yield %, Figure S2: Experimental domain of the FFD and CCD for the variables irradiation power and extraction time. The dot line represents the maximum power values applicable by the microwave extractor, Figure S3: CCD Residuals analysis for yield (upper left panel). thymol (upper right panel). γ-terpinene (lower left panel) and p-cymene (lower right panel) content, Figure S4: Pearson correlation between all the responses accurately modelled by the CCD, Figure S5: Effect of the milling process on the size and roundness of ajowan schizocarps, Figure S6: Images of the crystals isolated from the ajowan EO (on the left) and their 1H NMR spectrum, Table S1: FFR Coefficient analysis for yield (in coded unit), Table S2: CCD coefficient analysis for yield and thymol, γ-terpinene and p-cymene (lower right sub-table) content reported as coded variables, Table S3: Chemical composition of the ajowan essential oils obtained by HD (HDV1 and HDCONV) and MAE (EOPT and EV1).
