# Supplementary material for: A Design of Experiment (DoE) Approach to Model the Yield and Chemical Composition of Ajowan (Trachyspermum ammi L.) Essential Oil Obtained by Microwave-Assisted Extraction
Source: Pharmaceuticals (Basel). 2021 Aug 19;14(8):816. doi: 10.3390/ph14080816 (PMC8398832; doi:10.3390/ph14080816)
Supplement: Supplementary file 1 [file pharmaceuticals-14-00816-s001.zip › pharmaceuticals-1334628-supplementary.pdf]

Supplementary information

of

A design of experiments (DoE) approach to model the yield and chemical composition of ajowan (Trachyspermum ammi L.) essential oil obtained by microwave-assisted extraction

Eugenia Mazzara[a], Serena Scortichini[b], Dennis Fiorini[b], Filippo Maggi[a], Riccardo Petrelli[a], Loredana Cappellacci[a], Giuseppe Morgese[a], Mohammad Reza Morshedloo[c], Giovanni Filippo Palmieri[a], Marco Cespi[a*]

[a]School of Pharmacy, University of Camerino, 62032 Camerino, Italy

[b]School of Science and Technology, University of Camerino, 62032 Camerino, Italy

[c]Department of Horticultural Science, Faculty of Agriculture, University of Maragheh, 55181 83111, Iran

*Corresponding author: Marco Cespi (marco.cespi@unicam.it)

Table S1: FFR Coefficient analysis for yield (in coded unit)

Term	Effect	Coef	SE Coef	T-Value	P-Value	VIF
Constant		2.4507	0.0515	47.59	0.000	
Microwave power (w/g)	0.2091	0.1045	0.0515	2.03	0.073	1.00
Extraction time (min)	1.4136	0.7068	0.0515	13.73	0.000	1.00
Water-to-seed ratio (%)	0.1734	0.0867	0.0515	1.68	0.126	1.00
Preliminary moistening time (h)	0.0879	0.0440	0.0515	0.85	0.415	1.00
Preliminary milling process	0.2138	0.1069	0.0515	2.08	0.068	1.00
Extraction cycle	-0.2400	-0.1200	0.0515	-2.33	0.045	1.00

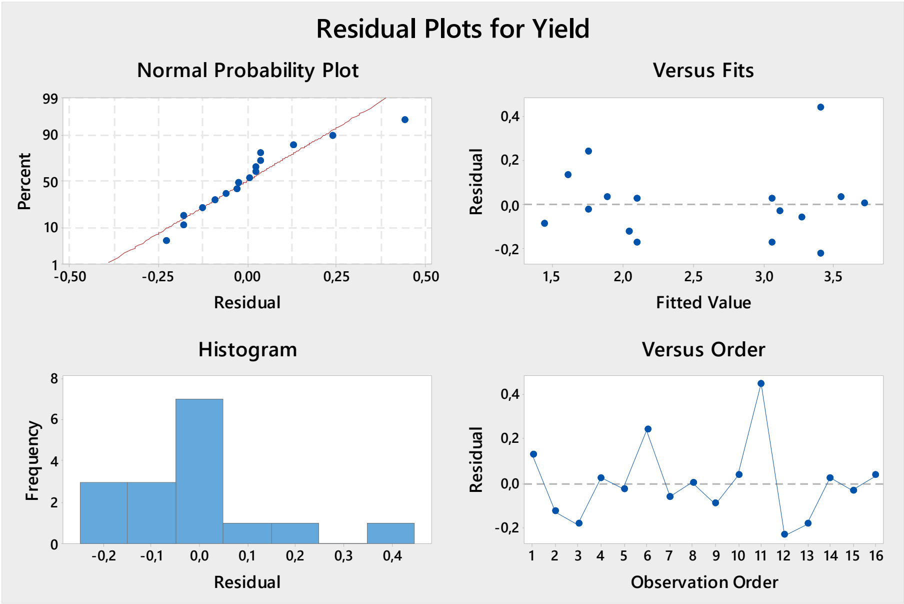

Figure S1: FFR Residuals analysis of yield %.

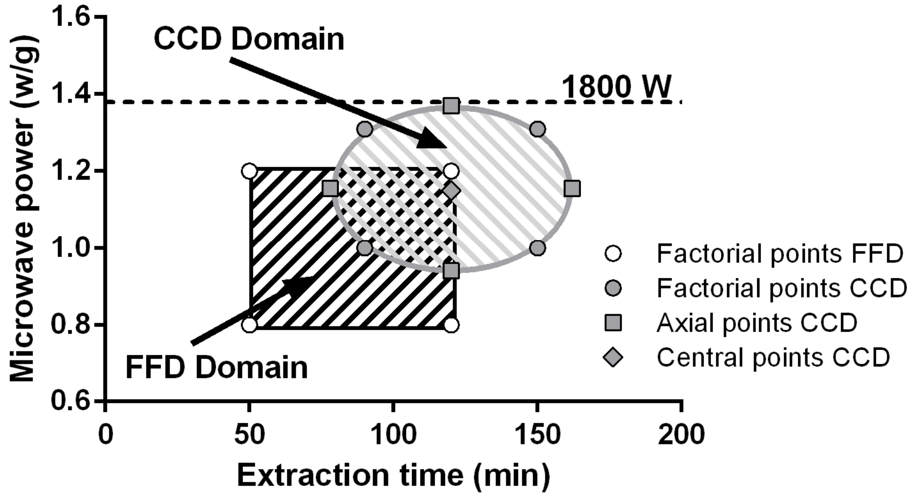

Figure S2: Experimental domain of the FFD and CCD for the variables irradiation power and extraction time. The dot line represents the maximum power values applicable by the microwave extractor.

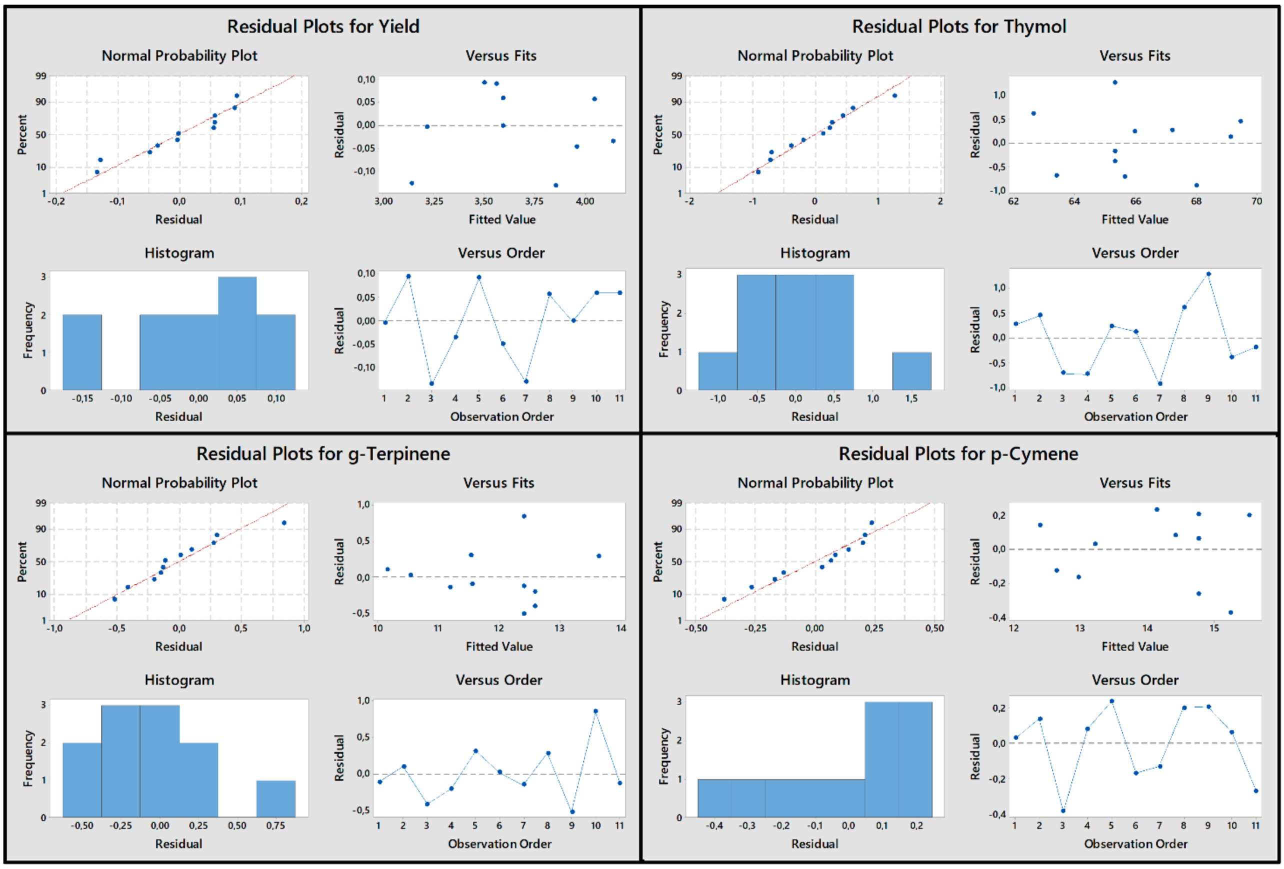

Figure S3: CCD Residuals analysis for yield (upper left panel). thymol (upper right panel). γ-terpinene (lower left panel) and p-cymene (lower right panel) content.

Table S2: CCD coefficient analysis for yield and thymol, γ-terpinene and p-cymene (lower right sub-table) content reported as coded variables. The abbreviation used are: MP, microwave power; ET, extraction time.

	Term	Coef	SE Coef	T-Value	P-Value	VIF
Yield	Constant	3.5918	0.0406	88.55	0	
	MP	0.141	0.0341	4.13	0.004	1
	ET	0.3202	0.0341	9.38	0	1
	MP^2	0.0838	0.0388	2.16	0.068	1
	**Term**	**Coef**	**SE Coef**	**T-Value**	**P-Value**	**VIF**
Thymol	Constant	65.313	0.332	196.56	0	
	MP	1.124	0.28	4.02	0.005	1
	ET	-1.905	0.28	-6.81	0	1
	MP^2	1.112	0.318	3.5	0.01	1
	**Term**	**Coef**	**SE Coef**	**T-Value**	**P-Value**	**VIF**
γ-terpinene	Constant	12.401	0.205	60.62	0	
	MP	-0.35	0.172	-2.03	0.088	1
	ET	0.867	0.172	5.04	0.002	1
	MP^2	-0.686	0.196	-3.5	0.013	1
	MP* ET	0.35	0.243	1.44	0.201	1
	**Term**	**Coef**	**SE Coef**	**T-Value**	**P-Value**	**VIF**
p-cymene	Constant	14.753	0.155	95.4	0	
	MP	-0.415	0.0947	-4.38	0.005	1
	ET	1.0192	0.0947	10.76	0	1
	MP^2	-0.603	0.113	-5.35	0.002	1.09
	ET^2	-0.341	0.113	-3.02	0.023	1.09

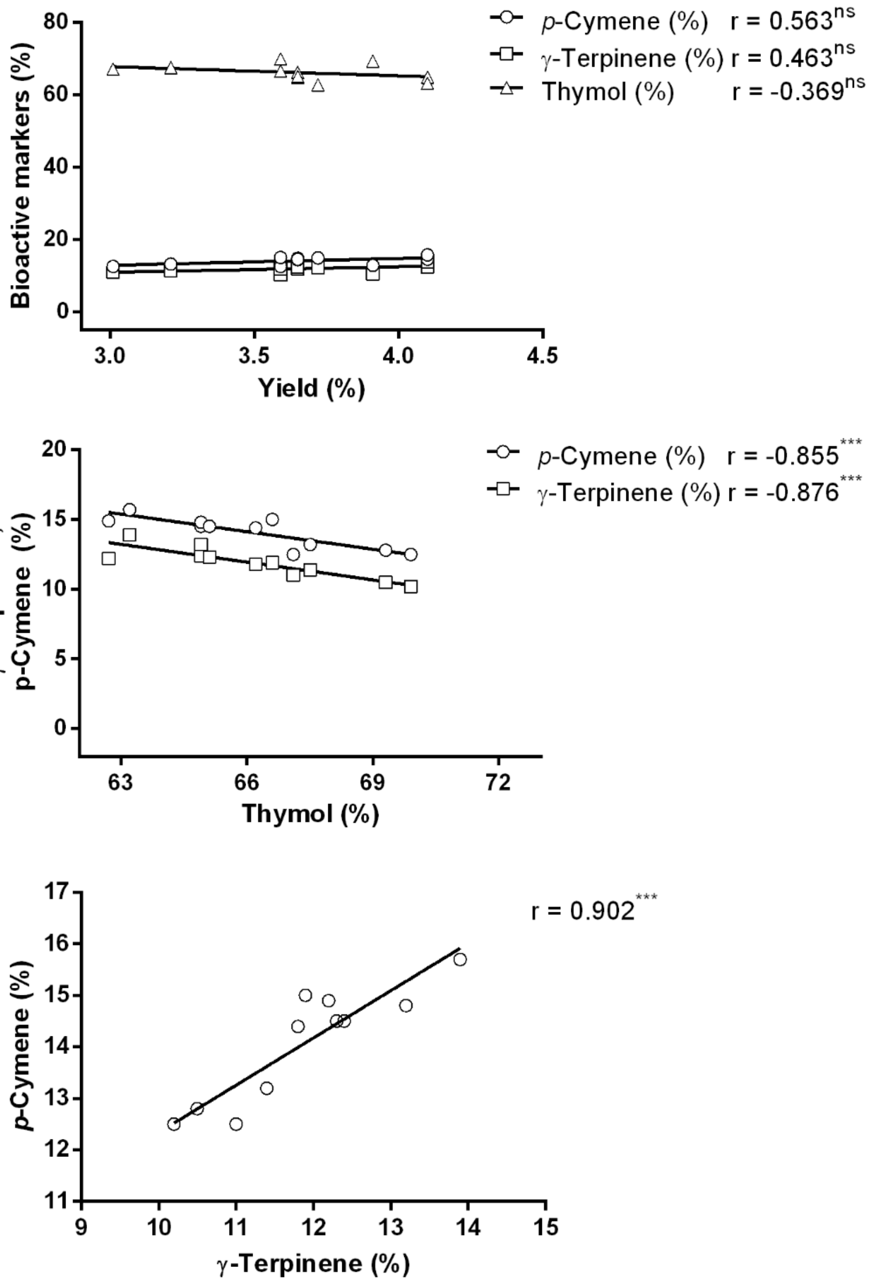

Figure S4: Pearson correlation between all the responses accurately modelled by the CCD.

Table S3: Chemical composition of the ajowan essential oils obtained by HD (HD_{V1} and HD_{CONV}) and MAE (E_{OPT} and E_{V1})

N°	Component[a]	RI[b]	RI LIT.[c]	Relative peak area (%)				ID[d]
				MAE		HD		
				E_{OPT}	E_{V1}	HD_{V1}	HD_{CONV}	
**1***	**α-thujene**	**920**	924	0.1	0.2	0.1	0.2	RI.MS
2*	α-pinene	926	932	tr	tr	tr[e]	tr	RI.MS
3*	sabinene	965	969		tr	tr		RI.MS
4*	β-pinene	967	974	0.4	0.6	0.6	0.8	RI.MS
5*	myrcene	989	988	0.1	0.2	0.1	0.1	RI.MS
6*	α-terpinene	1014	1014	tr	0.1	0.1	tr	RI.MS
7*	*p*-cymene	1022	1021	15.6	18.1	18.2	21.8	Std
8*	β-phellandrene	1025	1024	0.2	0.2	0.3	0.3	RI.MS
9*	γ-terpinene	1055	1054	11.5	12.6	14.0	16.8	Std
10**	trans sabinene hydrate	1095	1098		0.1	tr		RI.MS
11**	terpinen-4-ol	1173	1174		tr	tr		RI.MS
12**	thymol	1296	1289	70.2	66.6	65.4	57.5	Std
13**	carvacrol	1301	1298	1.3	1.2	1.0	1.2	Std
	Total identified (%)			99.3	99.8	99.9	98.6	
	Grouped compounds							
	Monoterpene hydrocarbons* (%)			27.9	31.9	33.5	40.0	
	Oxygenated monoterpenes** (%)			71.5	67.9	66.4	58.6	

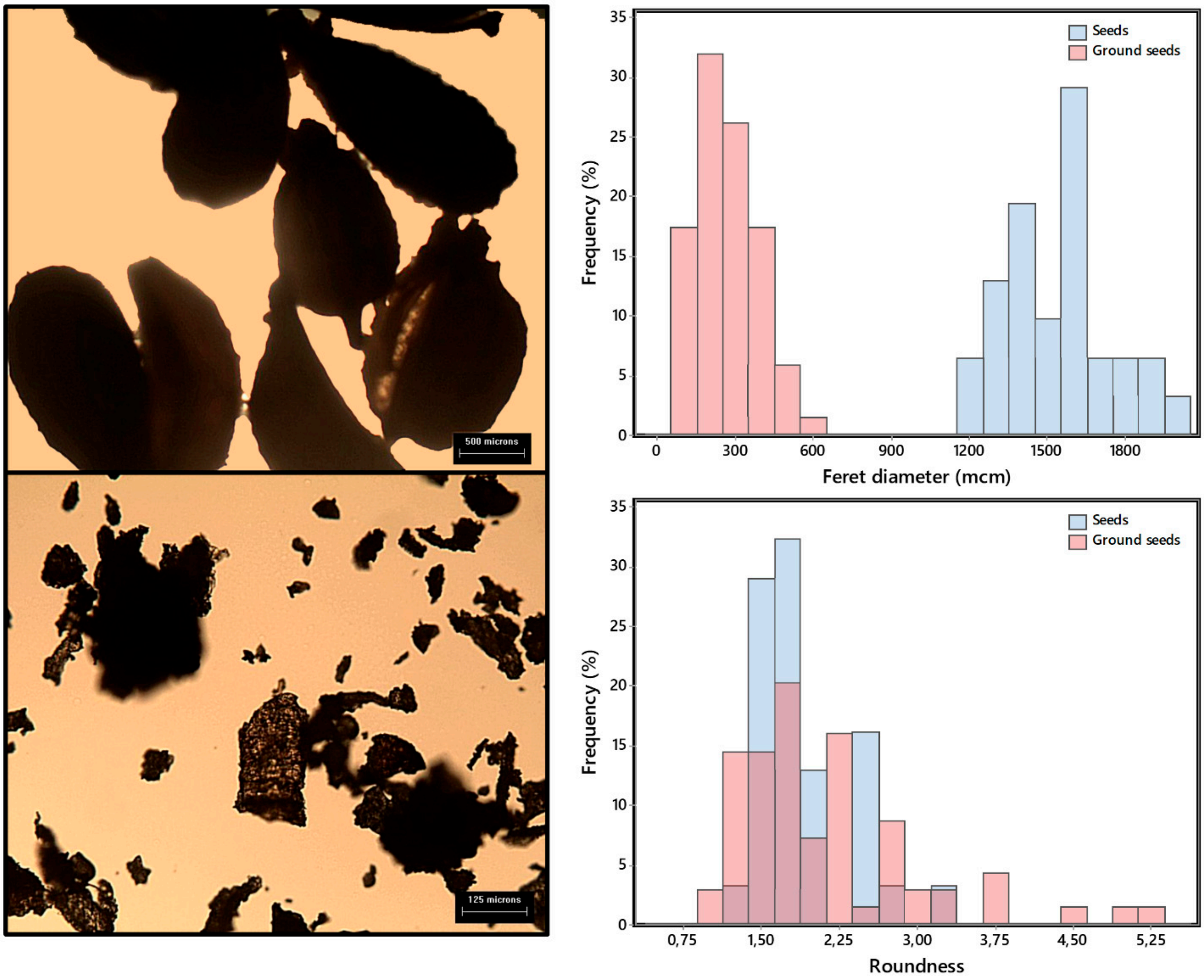

Figure S5: Effect of the milling process on the size and roundness of ajowan schizocarps. The microscopic pictures of schizocarps as received and grounded are reported in the upper and lower left panel respectively, while the particle size and the roundness values distributions of grounded and un-grounded schizocarps are reported in the upper and lower right panels respectively. Pictures were acquired using an optical microscope (MT9000, Meiji Techno Co. Ltd, JP) equipped with a 3-megapixel CMOS camera (Invenio 3S, DeltaPix, DK). The objective lens were a 2.5X and 10X for grounded and un- grounded schizocarps respectively. Size and roundness analysis were carried out using image analysis software (Image Pro Plus, Media Cynernetics Inc.). The size reported refers to the mean Feret diameter. The particles size distributions were characterized by the following values (μm): D50 of 1554 and IQR of 247 for ungrounded seeds, D50 of 253 and IQR of 180 for grounded seeds. The roundness distributions were characterized by the following values: median of 1.71 and IQR of 0.49 for ungrounded seeds, median of 1.86 and IQR of 0.92 for grounded seeds.

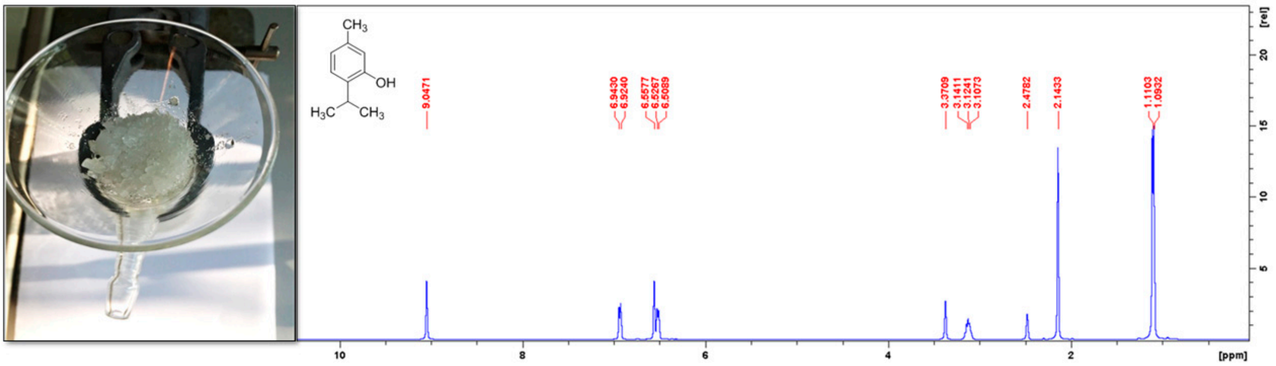

Figure S6: Images of the crystals isolated from the ajowan EO (on the left) and their ^{1}H NMR spectrum.
